# Noise and vestibular perception of passive self-motion

**DOI:** 10.3389/fneur.2023.1159242

**Published:** 2023-04-26

**Authors:** Francesco Lacquaniti, Barbara La Scaleia, Myrka Zago

**Affiliations:** ^1^Laboratory of Neuromotor Physiology, IRCCS Santa Lucia Foundation, Rome, Italy; ^2^Department of Systems Medicine, Centre of Space Bio-medicine, University of Rome Tor Vergata, Rome, Italy; ^3^Department of Civil Engineering and Computer Science Engineering, Centre of Space Bio-medicine, University of Rome Tor Vergata, Rome, Italy

**Keywords:** hair cells, vestibular afferents, central vestibular pathways, vestibular thresholds, galvanic stimulation, stochastic resonance, vestibulopathies, vestibular rehabilitation

## Abstract

Noise defined as random disturbances is ubiquitous in both the external environment and the nervous system. Depending on the context, noise can degrade or improve information processing and performance. In all cases, it contributes to neural systems dynamics. We review some effects of various sources of noise on the neural processing of self-motion signals at different stages of the vestibular pathways and the resulting perceptual responses. Hair cells in the inner ear reduce the impact of noise by means of mechanical and neural filtering. Hair cells synapse on regular and irregular afferents. Variability of discharge (noise) is low in regular afferents and high in irregular units. The high variability of irregular units provides information about the envelope of naturalistic head motion stimuli. A subset of neurons in the vestibular nuclei and thalamus are optimally tuned to noisy motion stimuli that reproduce the statistics of naturalistic head movements. In the thalamus, variability of neural discharge increases with increasing motion amplitude but saturates at high amplitudes, accounting for behavioral violation of Weber’s law. In general, the precision of individual vestibular neurons in encoding head motion is worse than the perceptual precision measured behaviorally. However, the global precision predicted by neural population codes matches the high behavioral precision. The latter is estimated by means of psychometric functions for detection or discrimination of whole-body displacements. Vestibular motion thresholds (inverse of precision) reflect the contribution of intrinsic and extrinsic noise to perception. Vestibular motion thresholds tend to deteriorate progressively after the age of 40 years, possibly due to oxidative stress resulting from high discharge rates and metabolic loads of vestibular afferents. In the elderly, vestibular thresholds correlate with postural stability: the higher the threshold, the greater is the postural imbalance and risk of falling. Experimental application of optimal levels of either galvanic noise or whole-body oscillations can ameliorate vestibular function with a mechanism reminiscent of stochastic resonance. Assessment of vestibular thresholds is diagnostic in several types of vestibulopathies, and vestibular stimulation might be useful in vestibular rehabilitation.

“It would be a dull, gray world without noise” (1).

## 1. Introduction

The vestibular system monitors all 6 directions of head motion, 3 translations and 3 rotations. Rotational movements are sensed by 3 roughly orthogonal semicircular canals, while gravito-inertial accelerations are sensed by two otolith organs (the utricle and the saccule). In concert with vision and proprioception, the vestibular system contributes to the perception of head position, orientation and displacement, in addition to controlling body posture, head and eye movements (2). When experimentally tested with repeated trials, vestibular perception of passive whole-body motion is quantified in terms of accuracy (systematic or average inter-trial error) and precision (trial-to-trial variability) of the subjective responses to the motion stimuli (3). Vestibular motion perception depends on both stimulus characteristics and noise (4).

In this review, we consider how various sources of noise impact on the neural processing of self-motion signals. But what is noise and how does it affect sensory processing? The answer is not as trivial as it would appear *prima facie*.

### 1.1. Definition of noise

In information theory, noise represents random, unpredictable perturbations of the transmitted signal (5). Specifically, noise is any form of interference that has the potential to alter a signal as it travels from a transmitter to a receiver, or within the receiver itself. The statistical properties of noise are defined by its power spectral density S(f). For instance, white noise has equal power in any band of a given bandwidth, i.e., S(f) = constant. The term of colored noise is used to refer to any non-white noise. Thus, pink noise has power spectral density that decays proportionally to frequency S(f) = 1/f, and Brownian noise has S(f) = 1/f^2^. The general power spectral density dependence on frequency is expressed as S(f) = constant/f^α^, with 0 < α < 2 (6).

We further distinguish intrinsic (or endogenous) from extrinsic (or exogenous) noise (7). The former refers to the stochastic fluctuations within the system under consideration, caused by the inherently probabilistic nature of the underlying processes. The latter refers to the stochastic processes outside the system.

#### 1.1.1. Intrinsic noise

Intrinsic noise is ubiquitous at all levels of the vestibular system, from the peripheral apparatus in the inner ear to the neural networks in the brain. It can arise as a consequence of random fluctuations in thermodynamics, quantum mechanics, fluid mechanics, mechano-electrical transduction and amplification at the hair cells, membrane excitability, opening and closing of ion channels, synaptic transmission, spike timing, postspike recovery of excitability, or network connections at all stages of the vestibular pathways (8–11).

In particular, neuronal noise designates random influences on the transmembrane voltage of single neurons and, by extension, on the firing activity of neural networks (12–14). This noise can affect the transmission and integration of signals from other neurons, as well as modify the firing activity of neurons in isolation.

Several neural phenomena exhibit 1/f^α^ power spectral density (see section 1.1). Examples are represented by the ion channel noise in neurons, the time density fluctuations of action potentials in the squid giant axon, the activity of ensembles of neurons in the human brain recorded from relaxed subjects by magnetoencephalography (6).

#### 1.1.2. Extrinsic noise

Extrinsic noise is imposed by the fluctuating environment in which the body is immersed. For instance, it is represented by naturally occurring mechanical perturbations of the head and body, such as those occurring during a bus or metro ride. Extrinsic noise can also be applied during experimental or clinical manipulations to stimulate the vestibular system. This is the case of the random mechanical vibrations of the head and body (15, 16), air-conducted sound associated with loud clicks or tone bursts (17, 18), bone-conducted vibrational waves (17, 19, 20), percutaneous application of galvanic currents at the mastoids (21, 22), irrigation of cold or warm water or gas injection into the external auditory canal (23).

### 1.2. Noise or signal?

There is an important caveat in the discussion of noise versus signal processing. In contrast with electronics and artificial communication systems, in neurobiology noise is not always distinguishable from the signal, or it may even be part of the signal. Noise in one system may be considered the signal or dynamics in another system, or at a different spatiotemporal scale (13).

Variability of interspike timing (time intervals between action potentials) results from the inevitable effects of generating spikes by sensory or synaptic processes, and is a major source of intrinsic noise. However, neural discharge variability also represents an important part of the signal that is transmitted to other neurons. It becomes a critical component of the neural code by increasing information transmission (14, 24).

As for non-neural factors, head oscillations associated with daily activities, such as walking, running, going up/down the stairs, bus or metro rides, etc. result from the combined effects of environmental perturbations and neuromechanical responses (see section 7). They thus include both extrinsic noise and signals to the brain, and in particular the vestibular system (25–28).

### 1.3. Noise: A hindrance or a benefit?

The neural and behavioral consequences of noise can be opposite, resulting in a hindrance or an advantage depending on the context. Random fluctuations and disturbances limit the efficiency of biological networks across a wide spectrum of scales, by obscuring the signals or by interfering with the transfer of information (11; (29)). Irrespective of whether the source is intrinsic or extrinsic, the presence of noise can be disruptive by decreasing sensory precision and/or accuracy (30–33).

Although neural variability may augment from the sensory periphery to central neurons due to strong synaptic bombardment, there exist multiple mechanisms in the brain that tend to mitigate the impact of noise (8). For instance, both filtering and network topologies capable of suppressing colored fluctuations represent examples of noise-cancelling strategies (29).

Noise can also be a source of variability that cells exploit advantageously (13, 14, 34). First, variability of neural discharge (interspike time intervals) may depend on stimulus amplitude, increasing for low-amplitude stimuli but saturating at high-amplitude values (35). Second, the high variability of discharge displayed by central vestibular neurons may prevent phase-locking or entrainment (14, 36). Thus, under natural conditions, the vestibular system uses increased variability to promote fidelity of encoding by single neurons (37). Third, tuning properties of some vestibular neurons is optimally matched to the statistics of naturalistic noisy inputs from the environment (38). During brain development, internal models of physics encoded in spontaneous neural activity become gradually adapted to the statistical structure of the natural sensory environment (39). As a result, perceptual performance – which is the final output of sensory processing – can be exceedingly precise despite, or in some cases thanks to, the presence of some amount of noise.

One should further consider that variability of neural discharge contributes to noise in a very different way depending on whether noise is combined linearly (additive noise) or nonlinearly (multiplicative noise) with the signal. Thus, in a linear system, resting discharge variability contributes directly to trial-to-trial variability to repeated stimulus presentations. However, this is no longer true in a nonlinear system (40). This is why irregular vestibular afferents, which have higher resting discharge variability than their regular counterparts, display lower trial-to-trial variability during stimulation, leading to increased information transmission *via* spike timing than regular afferents ((41, 42); see section 4).

Not only can the nervous system limit the negative impact of noise on signal transmission, but it can actually draw direct benefits from the presence of noise. There is now ample evidence that noise can lead to a paradoxical enhancement of neural sensitivity under specific conditions (14). For instance, the addition of a given amount of noise to a subthreshold signal can jolt otherwise silent sensory neurons above their spiking threshold, a phenomenon known as subthreshold stochastic resonance [(43); see section 8]. Indeed, it has been argued that neural systems function reliably *because* they have evolved in the presence of noise (7, 11, 44, 45).

In this article, we provide a brief review of some effects of various sources of noise on the vestibular processing of passive self-motion cues, with a special emphasis on vestibular motion thresholds. Results from human and non-human primates will be mainly discussed. We will consider only marginally the effects of noise on the vestibular control of eye, head or limb movements. For these issues as well as for many others not covered here (e.g., active self-motion), the interested reader is referred to excellent reviews and books [e.g., (2, 9, 46–48)].

## 2. Self-motion perception and noise

### 2.1. Vestibular thresholds

Behaviorally, vestibular motion perception is quantified by means of the psychometric functions for detection or discrimination of head-centred translations, rotations or tilts (3). The spread of the psychometric function corresponds to the vestibular threshold, that is, the minimum amount of motion necessary to reliably detect motion or recognize motion direction. Thresholds vary inversely to precision, and reflect the contribution of both intrinsic and extrinsic noise to vestibular perception (49). Here, intrinsic noise is contributed by all sources of noise from mechano-electrical transduction by hair cells in the inner ear to central processing of the stimuli in the brain. Extrinsic noise is contributed by the motion platforms used to deliver the stimuli, which typically add small random vibrations to the motion waveforms (50, 51).

In general, the idea that sensory systems have fixed thresholds - below which a stimulus does not generate any percept - has long been abandoned. In fact, limits to the detectability of small stimuli are set by noise. In the absence of noise, there would be no threshold: arbitrarily small stimuli would generate proportionately small but nonzero responses (31). In the presence of noise, the threshold for an ideal observer is related to the distance between signal distribution (centred at mean signal) and noise distribution (centred at zero signal). According to signal detection theory (52, 53), the stimulus is a random variable and the perceptual decision derives from a comparison between a sample from this random variable and a predefined criterion. The difference *d*′ between noisy representations normalized by their standard deviations generally increases with stimulus strength. The shape of the psychometric function depends on the distribution of the noisy representation (52). Gaussian noise results in a psychometric function that is a cumulative Gaussian (3).

### 2.2. Multisensory contributions

In addition to vestibular cues, other sources of sensory information potentially contribute to passive self-motion perception (visual, auditory, cutaneous, muscular, visceral) (54). However, in a typical experimental setting to test motion direction discrimination, appropriate measures are taken to minimize non-vestibular cues, for instance by masking visual and auditory cues related to motion stimuli. In such case, the predominant role of the vestibular organs has been demonstrated by showing that the corresponding thresholds in human patients (55) or monkeys (56) with vestibular ablation are considerably degraded.

Motion perception typically involves more than one vestibular organ. Thus, head motion engages both otolith organs and semicircular canals, except for specific motion directions. In particular, rotations that do not change head orientation relative to gravity only engage semicircular canals, while interaural translations primarily engage the utricles, and craniocaudal translations engage the saccules. In the other cases, the brain integrates canal and otolith cues to determine self-motion more precisely than with either cue individually (57).

The otoliths sense gravito-inertial accelerations, and do not distinguish forces produced by changes in the orientation of the head relative to gravity from those produced during translational self-motion. In order to estimate head orientation, the brain integrates rotational head motion signals from the semicircular canals with gravito-inertial signals from the otoliths (2, 9). Integration of otolith and canal signals is based on an internal model of the effects of gravity and inertial motion, and involves the transformation from a head-centred to a gravity-centred reference frame (58–60).

In the light, vestibular cues are optimally combined with visual cues for self-motion discrimination (61–64), as well as for the perception of orientation relative to gravity (65, 66). Multisensory integration of self-motion cues can be explained based on probabilistic theories, such as Bayesian inference (46). The probability of a given heading direction can be derived by combining vestibular signals, optic flow, and prior knowledge, following Bayes’ rule. Each sensory source is weighted proportionally to its reliability (inverse of source variance or noise). By using prior knowledge about the expected structure of the signal and/or noise, sensory processing can compensate for the amount of noise (11, 67).

Bayesian models of self-motion perception address the problem of noise accumulation during inertial navigation. In theory, integration of vestibular signals at peripheral and central processing stages would result in the accumulation of errors over time (68). Since these errors do not typically occur, it has been posited that internal models of sensory dynamics and noise compute the probability distribution of motion variables by modeling velocity storage, for instance by using particle filtering (69, 70).

### 2.3. Vestibular pathways

Vestibular motion perception is the result of multistage processes (9). Sensory inputs are transduced by hair cells in the inner ear, which synapse on the vestibular afferents in cranial nerve VIII. The afferents project to the vestibular nuclei in the brainstem. From there, the central pathways involved in self-motion perception comprise the thalamus and vestibular cortical areas (47, 67). Noise enters at all stages of vestibular processing.

## 3. Noise at hair-cell transduction

Hair-cell mechanoceptors of different sensory organs (vestibular, auditory, vibratory, lateral line) operate over different frequency ranges, but they all use similar transduction mechanisms (9). In the absence of noise, hair-cell responses would be limited only by the physical constraints on the stimulus. The energy supplied to each hair cell at the human behavioral threshold is of the same order of magnitude as that of thermal motion (10). Sensitivity of hair cell bundles as mechanical detectors is very high (higher for auditory than vestibular cells), and the hair-cells responses are very fast (71). A hair cell is stimulated when the bundle of the cilia is deflected toward its tall edge (the kinocilium).

Since the cell makes instantaneous measurements of the position of the cilia, in theory, broadband voltage changes should be affected by at least the total displacement noise of the ciliary bundle. However, measurements show that the signals that are reliably detected from single hair cells can be considerably smaller than the broadband ciliary displacement noise, indicating that the hair cells filter the mechanical signal (31). Moreover, random noise due to the transduction of Brownian motion is reduced as each hair cell averages its input over an increasing period of time, since the cell typically receives many cycles of the stimulus (10). Averaging can mitigate the effects of noise also when several cells carry the same signal and each cell is affected by independent sources of noise (11).

**Figure 1:**
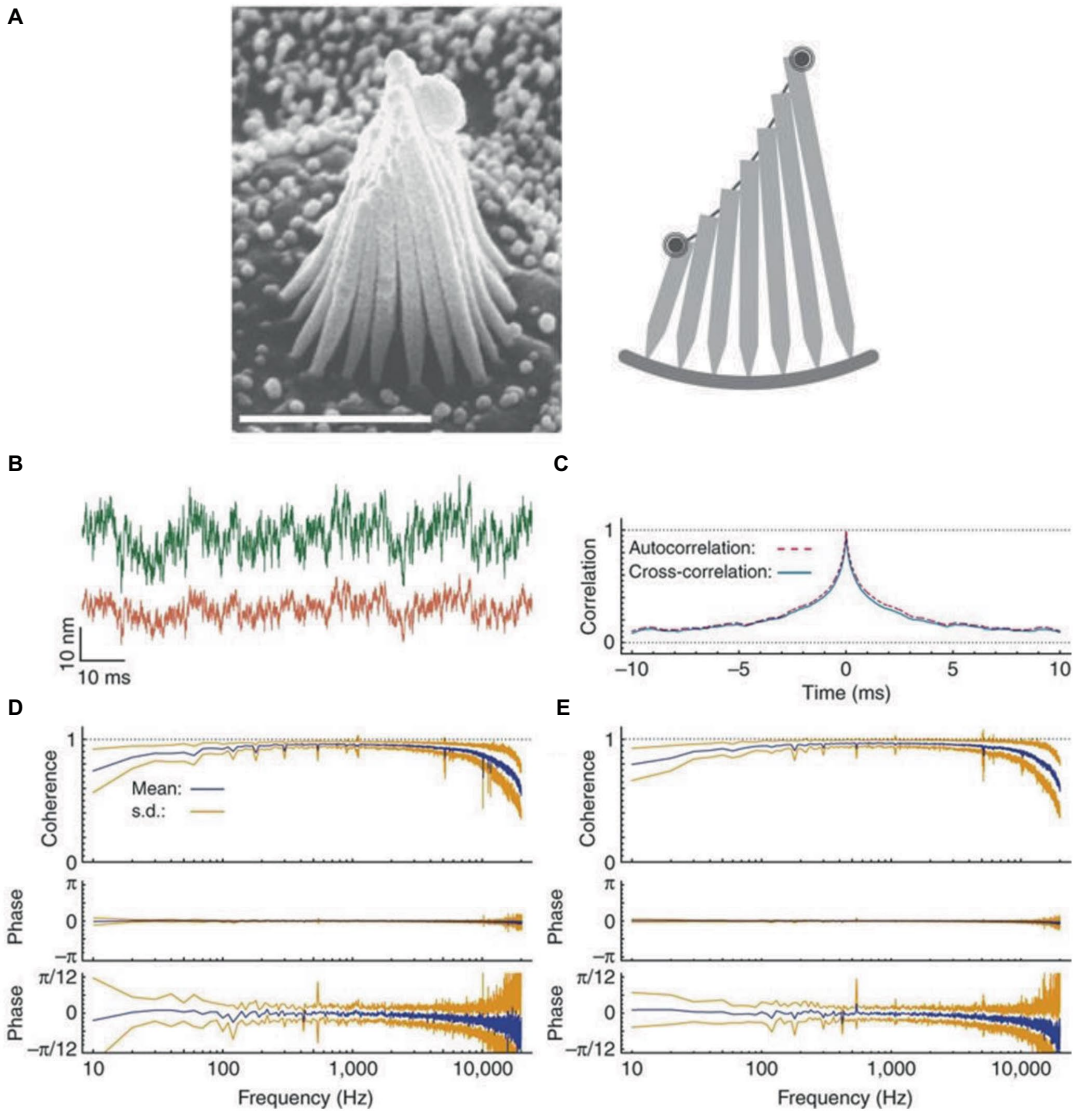
Coherent motion of stereocilia of hair cells of bullfrog’s sacculus. **(A)** Left: scanning electron micrograph of a hair bundle. Right: schematic diagram of a slice along the hair bundle, with the two spots representing the laser beams employed in interferometric measurements positioned on the bundle’s opposite edges. **(B)** When the green (top trace) and red laser beams are focused on the opposite edges of a hair bundle, the two records of thermal motion are very similar, except for different amplitudes due to different stereociliary lengths. **(C)** Crosscorrelation averaged over 20 records (blue line) and superimposed on the corresponding autocorrelation (red dashed line). **(D)** Coherency spectrum from the opposite edges of 18 hair bundles shows high values and negligible phase lag at frequencies up to 10 kHz. The phase spectrum shown at a higher magnification (bottom trace) shows a systematic deviation of the mean phase lag from zero at frequencies close to the analog filters’ cutoff, due to anti-aliasing filters. **(E)** For comparison, the spectrum for the beams focused on the same edge of the hair bundle is highly similar to that in panel **(D)**. The results in this Figure are for quiescent hair bundles, but the results for oscillating hair bundles are very similar. Reproduced with permission from Kozlov et al. (72).

There is also a mechanical substrate for response averaging and noise suppression, since the stereocilia of hair cells are constrained to move together. In fact, thermal movements of stereocilia located at opposite edges of a hair bundle exhibit high coherence and negligible phase lag (Figure 1) (72). Coupling tends to average random fluctuations in the movement of individual stereocilia. This assures the concerted gating of transduction channels, with the result of maximizing the sensitivity of mechanoelectrical transduction and amplifying the responses. In the vestibular organ, coupling is strengthened by the accessory structures (cupulae or otolithic membrane in canals and otolith organs, respectively) that constrain cilia movements.

Another mechanism that tends to reduce the impact of noise is represented by the band-limited frequency responsiveness of hair cells, allowing to reject noise components outside the preferred frequency range (10). For instance, viscous drag acting on the bundles of saccular hair cells limits Brownian motion to relatively low frequencies (73, 74).

Brownian motion of hair cell bundles might also facilitate mechano-electrical transduction of hair cells under specific conditions. Thus, by measuring the transduction current signal-to-noise ratio in isolated hair cells of the frog sacculus, Jaramillo and Wiesenfeld (75) found that very small (2–3 nm) random displacements of the bundles provide an optimal noise level that increases transduction sensitivity to weak signals. Bundles’ displacements that are smaller or larger of the critical level do not enhance the transduction sensitivity. They argued that Brownian motion can increase the transition rates between the open and closed states of the transduction channels through its influence on the hair bundles. Increases of signal-to-noise ratio were also observed in multiunit recordings from the afferent fibers of semicircular canals of chickens in response to endolymph movement and consequent cupula deflections (76). Both sets of results have been accounted for on the basis of stochastic resonance phenomena (see section 8).

## 4. Noise at vestibular afferents

For the vestibular afferents, noise shows up as variability of discharge. In mammals, hair cells synapse on two types of afferents from cranial nerve VIII, so called regular units and irregular units (9, 77). Regular units mainly provide bouton-endings to cylindrical type II hair cells in the peripheral (extrastriolar) zone of each vestibular organ. Irregular units have larger axons and provide calix-endings on flask-shaped type I hair cells, as well as bouton-endings to type II hair cells in the central (striolar) zone.

### 4.1. Variability of discharge rates

**Figure 2:**
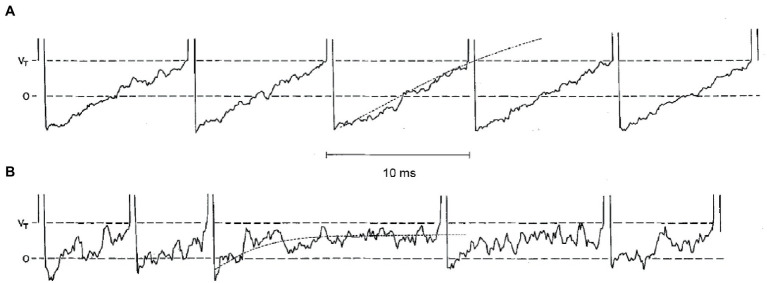
Simulations of five consecutive interspike voltage trajectories for a regular neuron in panel **(A)**, and for an irregular unit in panel **(B)**. Horizontal dashed lines are the resting potential (0) and the critical firing level (Vr). The dotted curves on the third interspike interval for each unit are the mean voltage trajectories. Reproduced with permission from Smith and Goldberg (79).

Both regular and irregular units possess a high resting discharge rate of action potentials (in the absence of any head motion), so that they can respond bidirectionally, increasing or decreasing the discharge rate as a function of motion direction. However, the variability (standard deviation) of interspike intervals is very small in regular units and very large in irregular units. Stochastic release of synaptic quanta results in synaptic noise that is responsible for the variability of interspike intervals. Simulations show that the spike timing precision depends on the small number of ion channels that are open at the action potential threshold (78). The resulting variability in spike timing is larger for weaker input signals, since the probability that the membrane potential crosses the spiking threshold depends more heavily on channel noise than for stronger inputs (11). The different regularity of discharge in vestibular afferents arises due to differences in synaptic noise and after-hyperpolarization (Figure 2) (79). In the regular units, after-hyperpolarization is deep and slow, and synaptic currents are sufficient for the average membrane voltage to cross the spiking threshold. In irregular units, quantal size and synaptic noise are larger and after-hyperpolarization is shallower and faster. These units require much less synaptic current to reach the same discharge rate as the regular units, but the voltage trajectory following the after-hyperpolarization does not reach the spiking threshold. As a result, their discharge is irregular because it is entirely governed by the random timing of synaptic quanta (79).

### 4.2. Coding properties

**Figure 3:**
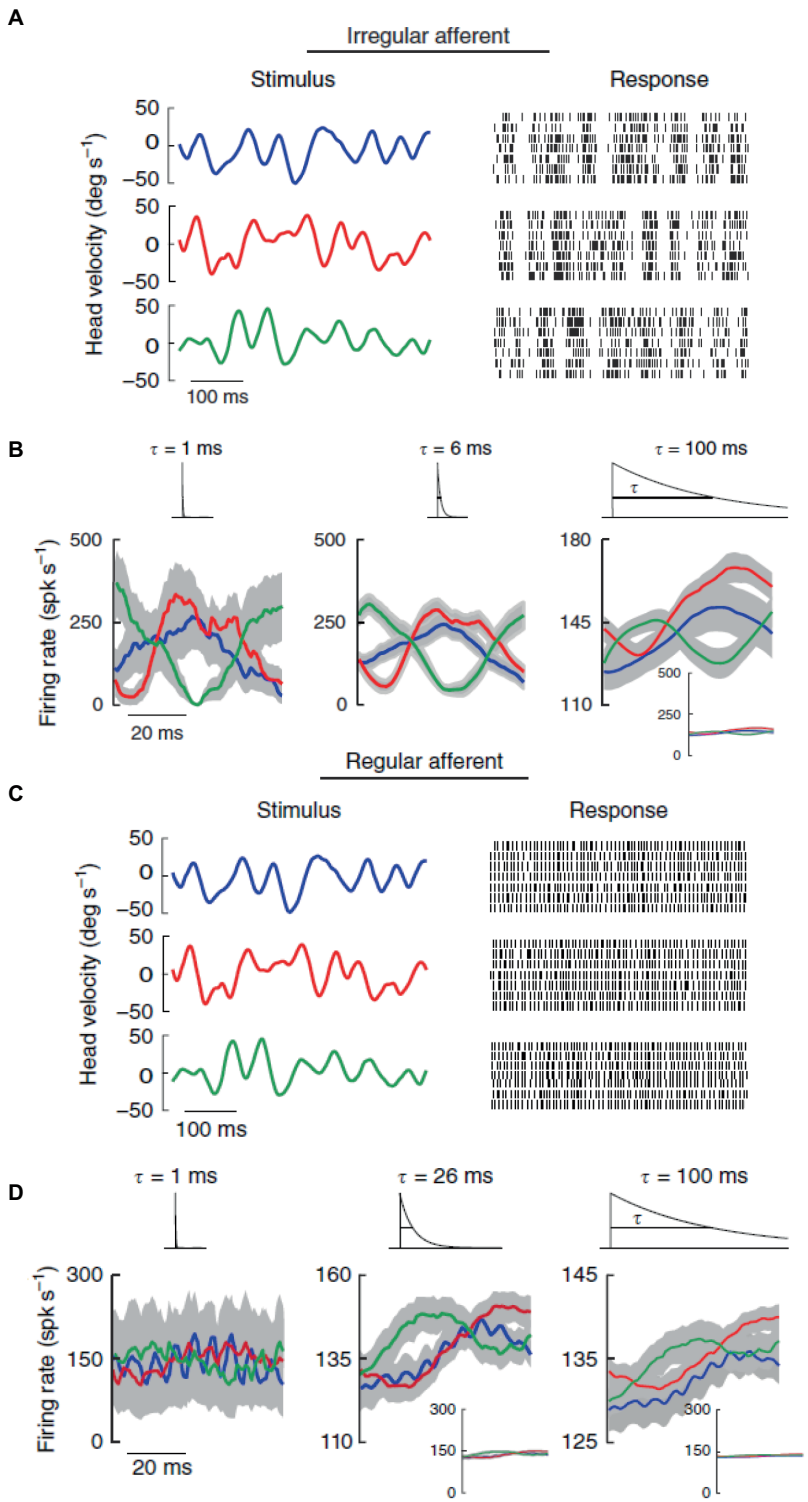
In contrast with regular afferents, irregular afferents discriminate between different stimulus waveforms through precise spike timing. **(A)** Spiking responses from an irregular afferent to repeated presentations of three different stimulus waveforms (broadband noise angular velocity stimulus, 20 Hz cutoff) with identical statistics (mean 0°/s, standard deviation 20°/s). **(B)** Average responses (standard error bands as shaded gray) of the irregular afferent to multiple repeated presentations to three different stimulus waveforms (red, blue, green) at 1 ms (left), 6 ms and 100 ms (right) timescales. The responses were obtained by convolving the spike trains with exponential kernels (top insets) that decay with these time constants. **(C)** Same as in panel **(A)** but for a regular afferent. **(D)** Same as in panel **(B)** but for the regular afferent. Reproduced with permission from Jamali et al. (42).

Different discharge variabilities are associated with distinctly different coding properties of vestibular stimuli by the two types of afferents, which then form two distinct channels for information transmission to higher brain centers (47, 77). Regular afferents transmit more information *via* firing rate, they tend to follow end-organ (canals or otoliths) dynamics, and have better detection thresholds especially for small-amplitude stimuli. By contrast, irregular afferents transmit more information *via* spike timing, and they are better tuned to stochastic naturalistic vestibular stimuli even of large amplitude. This has been shown for rotations stimulating the semicircular canals (Figure 3) (42), as well as for translations stimulating the otoliths (41). In both cases, broadband filtered white noise (20 Hz cutoff) was used, roughly mimicking naturally occurring head motions (see section 7).

At low frequencies of head motion stimulation (between about 0.1 and 10 Hz), both types of afferents respond roughly in phase with rotational velocity for canals and translational acceleration for otoliths (74, 80). At higher frequencies, both types of afferents have high-pass tuning such that neuronal response sensitivities increase with the frequency of head motion stimulation, but irregular afferents have considerably higher gains and phase leads than regular afferents (81).

### 4.3. Neurometric curves

In analogy to the psychometric curves determined with behavioral tests (see section 2.1), neurometric curves are constructed by fitting electrophysiological data as a function of the stimulus levels (82). Neural thresholds are computed as the standard deviation of the distribution of the neural responses. It is found that the precision of individual neurons in encoding head motion is considerably worse than the perceptual precision measured behaviourally [(80, 81); see however (83)]. Interestingly, neural thresholds of otolith afferents are independent of stimulus frequency and resting discharge regularity (80). This behavior originates from the parallel increment of trial-to-trial variability of discharge and neural sensitivity, such that their ratio remains constant (80), in contrast with what happens with the vestibular afferents from semicircular canals (81, 84).

By using motion stimuli resembling those encountered in naturalistic situations (25), it was found that, in contrast with single-afferent activity, the correlated activity between pairs of irregular afferents provides detailed information about the envelope of the head motion stimuli (85, 86). Modeling shows that such correlation-based coding of motion envelopes requires an optimal level of neural variability (86). This represents still another example of enhancement of information transmission due to noise inherent in neural responses (8).

## 5. Central processing in vestibular nuclei and ascending pathways

### 5.1. Vestibular nuclei

Most neurons of the vestibular nuclei integrate inputs from both otolith and canal afferents (9, 47). The main targets of projection in the vestibular nuclei are represented by position-vestibular-pause (PVP) neurons for the regular afferents and vestibular-only (VO) neurons for the irregular afferents (47). Detection thresholds for yaw rotation velocity of VO neurons are even higher than those of their afferent inputs, because VO neurons display greater variability (84). Therefore, ascending pathways from the vestibular nuclei must integrate information from populations of neurons to obtain perceptual performance levels comparable to those measured behaviourally (80, 84). Thus, estimated detection threshold at 1 Hz yaw rotation was about 4°/s for a pool of 12 VO neurons and about 2°/s for the same pool of regular afferents in macaque monkeys (84), compared to 0.5–1°/s threshold determined behaviourally in humans (87).

On the other hand, VO neurons nonlinearly integrate convergent afferent inputs (88), and are ideally tuned to respond to the envelopes of natural vestibular stimuli (25). Not only do VO neurons respond to the stimuli envelopes, but they also encode naturalistic self-motion statistics through stimulus whitening, i.e., by rendering the spectral power of the resulting neuronal response about constant (40). Naturalistic self-motion can be simulated in the laboratory by applying rotations with a time-course similar to that recorded while the monkey performs normal behaviors, such as walking or jumping (40, 89). Stimulus whitening is not inherited from their afferent neurons (since the latter do not display it), but results from a match between the input distribution on the one hand, the frequency spectrum of neural tuning and neural variability on the other hand (40), consistent with the matched-filter principle (90). Further improvements of tuning to naturalistic stimuli occur at later processing stages of the vestibular pathways (see below).

PVP neurons have lower resting discharge variability than VO neurons, and do not display stimulus whitening (89). However, they faithfully encode the motion stimuli waveforms, a critical function to generate the compensatory VOR eye movements observed during naturalistic self-motion stimulation (89).

### 5.2. Thalamus

As we noticed above, neural variability increases from the vestibular periphery to vestibular nuclei, but it may decrease at higher processing stages. This was found to be the case for neurons of the ventral posterior lateral (VPL) thalamus (35). VPL receives direct inputs from the vestibular nuclei and projects to multiple regions of the vestibular cortex, such as the parieto-insular vestibular cortex, ventral intraparietal area, area 2v of the intraparietal sulcus, and area 3a in the sulcus centralis (91). Carriot et al. (35) quantified how neural gain and variability of VPL neurons vary as a function of stimulus amplitude, and computed the neural discrimination thresholds for yaw rotations. They found that neural gain decreased with increasing stimulus amplitude, as expected. Strikingly, however, neural variability initially increased at low amplitudes but saturated at high amplitudes. By pooling the activities of multiple thalamocortical neurons, they obtained neurometric discrimination thresholds that matched the psychometric perceptual thresholds reported in behavioral studies (92, 93), as well as the violations of Weber’s law reported in the same studies. Therefore, vestibular discrimination thresholds estimated for both neurons and behavior are affected by neural variability, in addition to neural gain (35).

**Figure 4:**
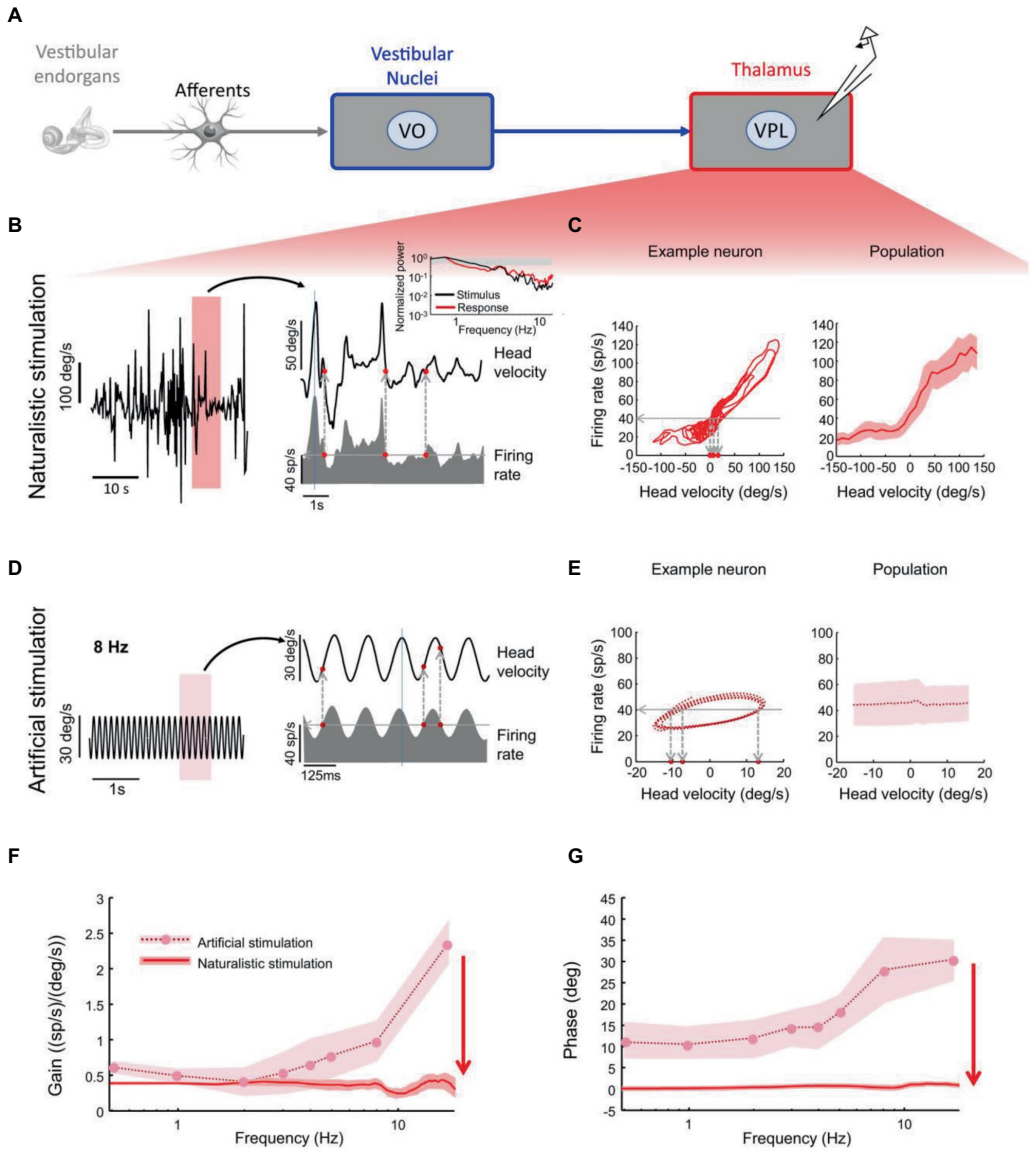
Vestibular thalamocortical neurons show reliable responses to naturalistic self-motion but not to artificial self-motion stimuli. **(A)** Schematic of peripheral and central vestibular pathways. Recordings are from the ventral posterior lateral (VPL) thalamus. **(B)** Left: Time series of a naturalistic self-motion stimulus. Right: Segment of this stimulus corresponding to the rectangle (top) and the firing rate response (bottom) of a VPL neuron. Inset: Stimulus (black) and spike train (red) power spectra from this neuron. Gray band: 95% confidence interval from a Poisson process whose power spectrum is independent of frequency. **(C)** Left: Firing rate versus head velocity for the neuron in panel **(B)**. Right: Population-averaged (*N* = 28) firing rate as a function of head velocity. **(D)** Left: Artificial sinusoidal (8 Hz) self-motion stimulus. Right: Segment this stimulus corresponding to the rectangle (top) and the firing rate response (bottom) from a VPL neuron. **(E)** Left: Firing rate versus head velocity for the neuron in panel **(D)**. The same firing rate (horizontal dashed line) can be elicited by multiple values of the head velocity (vertical dashed lines), which leads to ambiguity. Right: Population-averaged (*N* = 28) firing rate as a function of head velocity. **(F)** Population-averaged neural gain as a function of frequency for artificial (dashed) and naturalistic (solid) self-motion. **(G)** Population-averaged phase as a function of frequency for artificial (dashed) and naturalistic (solid) self-motion. Reproduced with permission from Carriot et al. (38).

VPL neurons projecting to cortex also demonstrate optimal encoding of naturalistic head motion stimuli (38). While these neurons ambiguously encode head velocity during artificial sinusoidal rotations, they faithfully encode head velocity during naturalistic noisy head rotations, with no significant phase lead across frequencies (Figure 4). In fact, during sinusoidal stimulation, a given firing rate (Figure 4E, left, horizontal line) was elicited by different instantaneous head velocity values (Figure 4E, left, vertical lines). By contrast, during noisy stimulation, for the same neuron a given firing rate (Figure 4C, left, horizontal line) was consistently elicited by similar instantaneous head velocity values (Figure 4C, left, vertical lines).

On the other hand, VO neurons of vestibular nuclei projecting to VPL still exhibit significant phase leads relative to head velocity during both artificial and naturalistic head rotations (38). In the context of a different sensory system (the cortical visual system of ferrets), tuning of neural activity patterns to the statistics of the natural environment has been shown to be a product of brain development (39). Following a Bayesian approach, this process has been interpreted as the maturation of an internal probabilistic model, statistical regularization being achieved *via* environmental interactions shaping neural activity based on prior expectations. It is likely that a similar process occurs also in the developing vestibular system.

### 5.3. MSTd

Liu et al. (94) compared the neurometric thresholds for horizontal heading discrimination of vestibular nuclei, cerebellar nuclei and cortical area MSTd (dorsal part of Medial Superior Temporal area). Subcortical neurons showed robust choice probabilities that exceeded those seen in area MSTd. The differences in the strength of choice-related modulations across brain areas depended to a large extent on the structure of interneuronal noise correlations. Correlated noise was measured between pairs of neurons, and was used as a proxy of information capacity of neural population codes. Significant choice probabilities were observed almost exclusively for neurons that responded selectively to translation, whereas neurons that represented net gravito-inertial acceleration did not show choice probabilities. The study suggests that reliable choice probabilities emerge in subcortical vestibular pathways by means of a signal transformation that distinguishes translation from orientation relative to gravity. A subsequent study showed that responses of all MSTd neurons during heading discrimination are selectively decoded relative to their vestibular heading preference, and selective decoding plays at least an important role in determining choice probabilities as correlated noise (95).

In sum, the precision of motion discrimination of single neurons as derived from neurometric functions is generally mediocre, but the global precision predicted by neural population codes matches the high perceptual precision derived from behavioral psychometric functions (see section 6). The number of neurons required in neural population models to match perceptual precision is much lower than the actual number of vestibular neurons, suggesting redundancy of encoding (47). The redundancy in the system has been explained by making reference to estimation theory: central neural processing of vestibular inputs is equivalent to an estimator that attempts to determine the estimand (physical stimulus) using multiple observations (69, 80).

## 6. Vestibular motion thresholds in humans

Vestibular thresholds for motion direction discrimination depend on stimulus characteristics (e.g., motion direction, amplitude, frequency), body orientation in space, and individual factors (e.g., age, pathology, medication). Here we give a concise account, and we refer the interested reader to Diaz-Artiles and Karmali (4) and Kobel et al. (96) for thorough reviews.

### 6.1. Perceptual precision

Vestibular motion discrimination is often tested by applying single cycle sinusoidal accelerations along a specific head-centered direction [e.g., (87, 97)], but other simple deterministic waveforms are also employed [e.g., (62, 98)]. Motion direction discrimination in young healthy adults can be very precise, thresholds in the dark approaching about 1°/s for head rotations (87), 1 cm/s for linear translations (99), 2° for static tilts relative to gravity (100), and 0.5–1°/s for dynamic roll tilt motions (57, 101). The thresholds for motion detection are even lower than those for direction discrimination, since detection but not discrimination can generally take advantage of vibratory cues associated with the motion stimulus (50). This is because humans are extremely sensitive to minute vibrations (102). Hair cells and vestibular afferents respond not only to fluid-conducted stimuli, but also to bone-conducted vibrational waves (especially irregular afferents) (17, 19, 20). However, mechanical vibrations can engage multiple receptors (auditory, cutaneous, muscular, visceral) in addition to the vestibular ones.

### 6.2. Relation with motion direction

Direction recognition thresholds may depend on motion direction. Thus, a previous study found that thresholds are lower for yaw rotations than for pitch or roll rotations (103). However, recent studies using modern psychophysical methodology did not find any significant difference between yaw, pitch or roll rotations (104, 105). A more consistent report is that thresholds are lower for inter-aural translations than naso-occipital or cranio-caudal translations (106, 107). Rotation thresholds may further depend on the orientation of the rotation plane relative to the anatomical alignment of the vertical canals (105). Multisensory integration of otolithic gravity cues with semicircular canal rotation cues enhances perceptual precision for tilt motions at frequencies below 2 Hz (105). Heading direction discrimination is more precise in the horizontal than the vertical plane of the head, and along the naso-occipital and inter-aural axes than intermediate axes (108). Heading thresholds depend on motion direction in head coordinates (see above) and on body orientation (better performance in the upright orientation), but do not depend on motion direction in world coordinates, demonstrating that the nervous system compensates for gravity (108, 109), although incompletely (110).

Higher perceptual sensitivity along specific motion directions can be reconciled with the neurophysiology of the vestibular organs (106, 108, 110). Thus, higher sensitivity along the inter-aural than cranio-caudal axis depends, at least in part, on the higher sensitivity of utricular afferents (responding to forces in the horizontal plane of the head) than saccular afferents [responding to forces in the sagittal plane, (111)].

### 6.3. Dependency on stimulus amplitude

Vestibular perceptual precision generally decreases with increasing amplitude of the stimulus. For instance, just-noticeable-difference (JND) increases with increasing speed of yaw rotation (about the Earth vertical) or horizontal translation (49, 92, 93). Increments of JNDs with increasing stimulus intensity is due to several factors, such as non-linearities of neural processing along the vestibular pathways, increase of neural noise with increasing stimulus amplitude (49), and probabilistic decision-making based on noisy signals (112). However, Weber’s law is not strictly obeyed in vestibular perception, motion discrimination thresholds tending to saturate for large stimulus amplitudes (92, 93). Improved discrimination performance for large stimuli can help to sense motion and maintain balance, even for strong, challenging perturbations. As we remarked before (see section 5.2), violation of Weber’s law has been accounted for also on the basis of the tuning of VPL neurons as a function of stimulus amplitude (35).

### 6.4. Dependency on stimulus frequency

Velocity thresholds for translation and rotation are consistent with a high pass filter, being relatively constant at about 1 Hz and above, and increasing with decreasing frequency below 1 Hz (87, 98, 113). In particular, velocity tilt thresholds increase in threshold up to about 1 Hz, but then decrease at 2 Hz (105). High-pass filtering presumably affects perceptual decision making, but not perceptual magnitude estimates (114). On the other hand, roll tilt displacement thresholds are consistent with a predominant positional cue at low frequencies (provided by otoliths) and a predominant velocity cue at high frequencies (provided by semicircular canals). Position tilt thresholds show a plateau below about 0.1 Hz and decrease with increasing frequency (55, 57).

### 6.5. Response variability

The specific values of the vestibular motion discrimination thresholds exhibit small session-to-session, intra-subject variability (115–117), unless discrimination is experimentally trained (see below). However, thresholds vary considerably (up to two orders of magnitude) across different healthy individuals of the same age (4, 107, 118). Attention, motivation, fatigue, and brain functional organization are examples of individual features that may affect the specific value of motion threshold for the same input stimuli (119). Individual motor skills also may affect motion thresholds. For instance, competitive gymnasts, with a long-term training in the maintenance of exquisite postural control, show superior pitch detection thresholds compared to control participants (120).

### 6.6. Conditioning and learning

Experimental exposure to conditioning passive motions can modify perceptual thresholds in a subsequent session. Thus, 10-min of conditioning stimuli consisting of high-amplitude stochastic yaw rotations (up to 300 deg/s^2^) increased yaw perceptual thresholds substantially (121). By contrast, 20-min of conditioning stimuli consisting of small-amplitude, subliminal interaural sinusoidal translations (2 cm/s^2^) significantly reduced the thresholds for motion discrimination along the same axis (122), as well as along the naso-occipital axis (123). Also, 5-days of intensive roll-tilt training ameliorated the corresponding discrimination thresholds as well as postural sway, if performance feedback was given after each trial (124). Presumably, both motor skills practice in experts (120) and perceptual training in non-experts (124) increase the signal-to-noise ratio of vestibular processing. However, the neural mechanisms underlying these perceptual improvements remain unknown. One possibility is that improvements result from an enhanced ability to integrate canal and otolith signals. Specifically, perceptual thresholds might be lowered by progressively reweighting different vestibular neural signals *via* Hebbian synaptic plasticity (124). The weight given to each source would progressively become proportional to its reliability (the inverse of the variance of the source).

### 6.7. Age dependency

On average, velocity motion thresholds do not depend on sex, but they depend on age (99, 107). Average thresholds do not change significantly with age between 18 and 40 years. However, after the age of 40, performance deteriorates systematically by about 15–83% per decade, the specific extent of decrement depending on motion direction (107) (see section 9.1 for potential substrates of these age effects).

### 6.8. Relation with VOR and posture

Since the JND for yaw rotations is not significantly different from the trial-to-trial variability of vestibulo-ocular reflex (VOR), it has been argued that noise sources inherent to the nervous system are shared by perceptual and VOR imprecision ((49); see however (125) for a different conclusion). A similar argument applies to the observed correlation between vestibular thresholds and postural stability. Higher thresholds tend to be associated with greater postural sway in young healthy people (101, 126). In elderly people, higher vestibular thresholds correlate with balance test failures (107). It has been hypothesized that postural sway arises from sensory noise, including vestibular, proprioceptive, and visual sources, in addition to motor noise (127).

## 7. Perceptual effects of externally applied noisy stimuli

As noticed above (see section 6.1), vestibular perceptual thresholds are typically tested with simple deterministic motion stimuli, such as sinusoidal stimuli. In this case, extrinsic noise is contributed by the random vibrations that motion platforms typically add to the motion stimuli [(50, 51), see section 2.1]. This kind of noise obviously does not represent the physical stimulus, and must be disentangled from the signal by the brain for the discrimination task.

### 7.1. Head oscillations under natural conditions

However, extrinsic noise does represent part of the physical stimulus processed by the vestibular system under most circumstances of daily life. Thus, the head routinely undergoes random displacements in all directions, for instance during standing posture, walking, running, going up/down the stairs, bus or metro rides, etc. These head displacements result from the combined effects of environmental perturbations (e.g., contact forces) and neuromechanical responses, the latter including voluntary movements, stabilizing reflexes, and body (head, neck, torso, limbs) biomechanics (25–28, 128). Interestingly, these head displacements provide both extrinsic noise and signals to the brain, since they contain information as to the specific behavioral context (i.e., they are different when walking vs. playing soccer).

Head oscillations have been quantified in different animal species (rodents, monkeys and humans) during normal activities typical of the animal species (25, 85, 129). These oscillations reach high intensities and contain a substantial power at high frequencies (up to 20 Hz). Moreover, they deviate from scale invariance (i.e., the power law dependency on frequency, S(f) = 1/f^α^) for both raw data (25, 129) and envelopes (85). Importantly, as we previously remarked (see sections 4 and 5), several vestibular neurons are less well tuned to deterministic sinusoidal stimuli than to broadband stochastic stimuli that reproduce natural head oscillations recorded during normal behaviours (40, 89). This has been shown to be the case for the irregular afferents of the vestibular cranial nerve (42), VO neurons in the vestibular nuclei (40), and neurons of the ventral posterior lateral (VPL) thalamus projecting to the vestibular cortex (38). The idea is that vestibular responses are molded on the natural biomechanical behavior of the head. Variability and tuning of specific populations of vestibular neurons complement the statistics of natural head motion stimuli (40). These neurons encode head velocity at all useful frequencies of stochastic motion stimuli much more faithfully than they do for sinusoidal stimuli [Figure 4; (38)].

### 7.2. Perceptual responses to mechanical perturbations

In view of the above background, it is interesting to consider also the perceptual responses to noisy external perturbations that roughly mimic natural conditions. The hypothesis is that some optimal level of random perturbations, entraining specific populations of central vestibular neurons, can enhance vestibular motion discrimination.

In humans, mechanical perturbations applied during vestibular motion discrimination tasks have been shown to increase, decrease or leave unaffected the thresholds, depending on the type and intensity of noise. Thus, wide-spectrum vibrations (1–500 Hz colored noise) applied directly to the mastoid did not affect the threshold for yaw rotation discrimination (130). Strong (about 140 cm/s^2^ peak acceleration) vertical whole-body oscillations (6 Hz) significantly increased (almost doubled) horizontal heading-direction thresholds (16). On the other hand, weak (between about 3 and 6 cm/s^2^ RMS) whole-body oscillations in the antero-posterior direction significantly decreased the thresholds of motion discrimination in the same direction as the perturbations (15).

**Figure 5:**
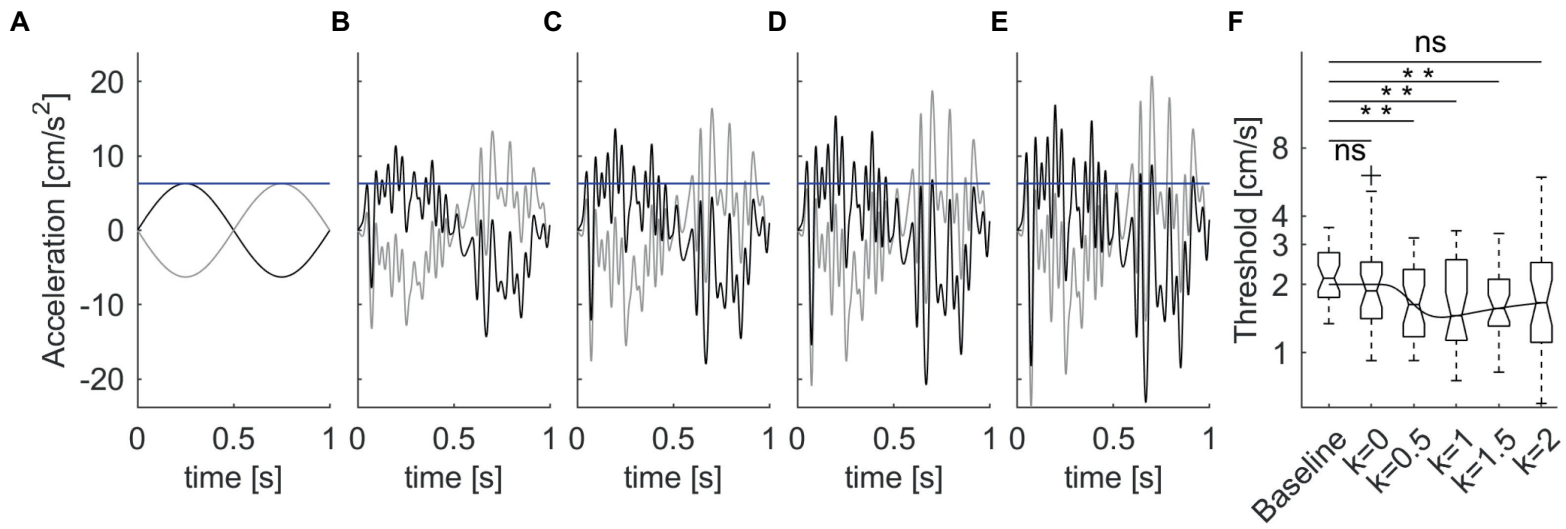
Enhancement of vestibular motion discrimination by stochastic whole-body perturbations. Sitting participants reported the perceived direction of antero-posterior 1 Hz sinusoidal motions. The amplitude of the stimulus was adjusted based on an adaptive staircase. **(A)**. Unperturbed stimuli in the forward (thick lines) and backward (thin lines) directions at baseline threshold (blue horizontal line) for a typical subject. **(B–E)** Perturbed stimuli with increasing noise amplitude [*k* = 0.5–2 in Equation 2, from panels **(B–E)**]. **(F)** Box-and-whisker plots of motion discrimination thresholds at population level (*N* = 30 participants). Overlaid line is the best fit of the population data with Equation 9 of stochastic resonance. Data from La Scaleia et al. (15).

In the latter study, the mechanical perturbations were added on top of single cycles of sinusoidal acceleration (Figures 5A–E). Perturbations consisted of bandpass (1.8–30 Hz) white noise ε(t) , scaled in amplitude as a function of the variance *δ^2^* of the acceleration stimulus corresponding to the individual threshold determined for each participant during the baseline condition, given the large intersubject variability of the baseline values (see section 6.5):


(1) $$\varepsilon^{*}\left(t\right)=\varepsilon \left(t\right)\sqrt{k\delta^{2}}$$

The proportionality constant *k* was 0, 0.5, 1, 1.5, or 2 in different blocks of trials (Figures 5A–E), presented in randomized order. The overall acceleration stimuli applied in the trials with noise were:


(2) $$a\left(t\right)=Asin\left(2\pi ft\right)+\varepsilon^{*}\left(t\right)$$

It was found that the threshold was non-linearly related to noise level (Figure 5F). The lowest thresholds (best performance enhancement) were obtained at low-to-intermediate noise levels, while thresholds with higher noise did not differ from baseline values without noise. However, there was considerable inter-subject variability of the optimal noise level and the extent of performance improvement. Participants with higher thresholds in the unperturbed conditions benefitted more (i.e., showed greater threshold improvements) from added noise than participants with lower unperturbed thresholds.

### 7.3. Perceptual responses to GVS

Galvanic vestibular stimulation (GVS) is frequently used to probe the vestibular system. GVS consists in electrical stimulation of the peripheral vestibular system obtained by placing an anodal electrode on the mastoid process behind one ear and a cathodal electrode behind the other ear (21, 22). The vestibular stimulation occurs without head motion. Noisy broadband GVS has been shown to ameliorate vestibular perception (131–135), as well as other vestibular-mediated responses such as ocular counter-rolling (136), balance (133, 137–139), vestibulo-spinal reflexes (135), mobility (140), locomotion (134, 141), and cross-modal visual perception (142). The effects on performance generally follow the nonlinear trend observed by (15) using mechanical noise, i.e., optimal GVS amplitude coincides with low-to-intermediate noise levels. As in the case of mechanical perturbations, also for GVS the optimal stimulus amplitude and the extent of performance improvement are quite variable across individuals [e.g., (137–139, 143)].

Optimal GVS amplitude has been shown to depend also on the individual level of vestibular function. Thus, greater noise amplitudes are generally required for individuals with reduced vestibular function due to aging (136) or bilateral vestibulopathy (143). However, persons with vestibular hypofunction improve more in response to GVS than normovestibular persons (143, 144).

As mentioned above, adding GVS on top of head motion stimuli can improve the thresholds of motion direction discrimination. Thus, GVS significantly decreased the thresholds for roll-tilt (131, 132) and inter-aural translation (145), but not the thresholds for yaw-rotation with the head pitched forward 71° (primarily resulting in stimulation of the semicircular canals), suggesting that GVS mainly affects otolith-mediated perception (146).

### 7.4. Neural substrates of GVS responses

In contrast with the hypothesis that GVS mainly affects the otolith system, an electrophysiological study in macaques showed that GVS generates robust activation of both canal and otolith primary afferents, with the result that neuronal detection thresholds in response to GVS are about constant across all afferents (147). This study also showed that the neural tuning of vestibular afferents differs drastically for GVS versus natural motion stimulation, due to the fact that GVS bypasses both the body biomechanics and the mechano-electrical transduction of the vestibular organs (147). This is because GVS directly activates the hair cells and vestibular afferents *via* electrical transmission (21). Since GVS determines simultaneous activation of primary afferents from all vestibular end organs on one side with concomitant inhibition of those on the contralateral side, the resulting stimulation pattern has no physiological motion equivalent (147). Moreover, GVS produces asymmetric activation of both semicircular canal and otolith afferents to the onset versus offset and cathode versus anode of applied current (148). In this respect, therefore, using mechanical noise instead of electrical noise to probe vestibular function may yield results more directly comparable with the natural behavior of the system.

## 8. Stochastic resonance and related phenomena

We have previously remarked that, depending on the context, noise can degrade or improve information processing and performance. In particular, there is now considerable evidence that a given amount of noise can lead to a paradoxical enhancement of neural sensitivity under specific conditions (see sections 1.2 and 3). In particular, the non-linear responses elicited by the application of either mechanical or galvanic noise (see section 7) are reminiscent of the phenomenon of subthreshold stochastic resonance, whereby an optimum level of random noise enhances the detection of weak signals (34, 43, 149, 150).

### 8.1. Classical stochastic resonance for periodic stimuli

Stochastic resonance was initially proposed in geophysics to account for the 100,000-year periodicity of the Earth ice ages (151, 152). According to this hypothesis, a weak periodicity in the insolation due to changes of Earth orbital parameters would determine regular transitions in the bistable energy potential describing long-term changes in global climate (151, 152). The concept and underlying formalism of stochastic resonance have subsequently found wide application to several neurophysiological phenomena subject to noise. We now summarize the key elements (34, 153).

The overdamped motion *x*(*t*) of a Brownian particle with a bistable potential in the presence of noise and periodic forcing is described by:


(3) $$dx\left(t\right)/dt=-dU\left(x\right)/dx+A_{0}\cos\left(\omega_{0}t\right)+\varepsilon \left(t\right)$$

where the trigonometric function represents a weak periodic forcing and *ε*(*t*) is random noise (intrinsic or extrinsic as in Equations 1, 2). *ε*(*t*) is classically Gaussian white noise (zero mean in the time-domain) and strength equal to *q*^2^ ( $k\delta^{2}$ in Equation 1). *U*(*x*) is the reflection-symmetric quartic potential driving the dynamics of the system:


(4) $$U\left(x\right)=\frac{x^{4}}{4}-\frac{\lambda x^{2}}{2}$$

This double-well potential has two minima located at $x_{\pm }=\pm \sqrt{\lambda }$ , separated by a potential barrier with height equal to:


(5) $$\Delta U\left(x\right)=\frac{\lambda^{2}}{4}$$

In the absence of periodic forcing, *x*(*t*) fluctuates around the local stable states with a statistical variance proportional to noise intensity. Noise-induced transitions are described by the Kramers rate equation of chemical kinetics (154), with transition time given by:


(6) $$\tau \left(q^{2}\right)=\frac{1}{\sqrt{2}\pi }\lambda e^{-\frac{\lambda^{2}}{2q^{2}}}$$

In the presence of periodic forcing, the reflection symmetry of the system is broken. The new potential *W*(*x*,*t*) becomes:


(7) $$W\left(x,t\right)=U\left(x\right)-A_{0}x\left(t\right)\cos\left(\omega_{0}t\right)$$

The periodic signal now biases the particle toward one or the other potential well, so that the particle jumps to the more globally stable well. For small amplitudes, the mean response of the system to the periodic signal is given by:


(8) $$x\left(t\right)=A\left(q^{2}\right)\cos\left(\omega_{0}t-\varphi \left(q^{2}\right)\right)$$

where *A*(*q^2^*) and ϕ (*q^2^*) represent, respectively, the noise dependent amplitude and phase lag:


(9) A(q2)=A0λq2τ(q2)τ(q2)2+ω024

(10) $$\varphi \left(q^{2}\right)=\arctan\left(\frac{\omega_{0}}{2\tau \left(q^{2}\right)}\right)$$

It has subsequently been shown that bell-shaped relationships captured by Equation 9 between the detection performance and noise can appear also with threshold-like systems (155) and in response to aperiodic signals (156).

### 8.2. Stochastic resonance in neurons

Stochastic resonance can arise in low-dimensional excitable systems, as shown for the Fitzhugh–Nagumo (FHN) neuron model. An optimal nonzero noise intensity maximizes the signal transmission in bistable FHN in response to either periodic or aperiodic inputs superimposed on a noisy background (8, 155). Stochastic resonance has also been found in higher-dimensional neuronal processes, due to the resemblance between interspike interval histograms and residence-time distributions of noise-driven bistable systems (34). Thus, stochastic resonance has been advocated for the observations that noise externally added to muscle spindles (157), cutaneous receptors (158) or vestibular hair cells (75, 76) enhances their responses to weak stimuli, which normally would be subliminal. Also, noise added to visual (159), auditory (160, 161), tactile (162) stimuli, or added directly to cortical networks (163) can improve the corresponding sensory thresholds.

Endogenous noise can also generate stochastic resonance (164). This was suggested to be the case for the Brownian motion of hair cell bundles and the enhancement of mechano-electrical transduction (see section 3) (75).

### 8.3. Perceptual effects of stochastic resonance

The effects of extrinsic noise on vestibular function have been reviewed above (see section 7). As a visualization aid to compare the results with stochastic resonance behavior, Equation 9 prediction is overlaid on the population results of vestibular motion discrimination in the presence of mechanical noise in Figure 5F (15). It has been argued that, at threshold, the perceptual discrimination of motion direction in a two-alternative forced-choice task [such as that of La Scaleia et al. (15)] involves a bistable potential well (see section 8.1): one well corresponds to perception of motion in one direction, and the other well perception of motion in the opposite direction (165). If the perceptual decision behaves like a Brownian particle, an optimal level of noise added to the system breaks the potential symmetry, and shifts the responses toward the correct direction with small physical stimuli. This would result in a stochastic resonance effect (165).

### 8.4. Stochastic facilitation

It has been proposed to use the more general term of stochastic facilitation to encompass biologically relevant noise benefits (24). This is because these benefits are not necessarily associated with frequency resonance in the system, and the responses may not strictly obey Equation 9 characteristic of classical stochastic resonance.

The presence or absence of stochastic resonance (or facilitation) in response to extrinsic noise depends not only on the amplitude of the extrinsic noise but also on that of the intrinsic one. Thus, stochastic resonance does not occur when the endogenous noise level is already equal to or higher than the optimal noise level for stochastic resonance (150). In this case, adding extrinsic noise would only degrade the detection performance. The influence of endogenous noise also explains why the optimal level of extrinsic noise and the extent of performance improvement are so variable across individuals [e.g., (15, 137–139, 143, 162, 163)]. As we previously remarked for the variability of vestibular motion thresholds (see section 6.5), neural noise can vary substantially as a function of individual factors, such as attention, motivation, fatigue, brain functional organization (11, 119).

In this regards, a long-known psychological phenomenon - the Yerkes-Dodson law - is closely reminiscent of stochastic resonance: a cognitive performance increases with physiological arousal, but only up to a point (166). When arousal becomes too high, performance decreases. However, both the cognitive performance and the arousal are quite variable across individuals.

Parallel summing arrays of noisy threshold elements can display another form of stochastic resonance, which occurs when the signal is predominantly suprathreshold (167). It has been shown that, in theory, this so-called suprathreshold stochastic resonance can lead to significantly greater signal enhancements than can be obtained using subthreshold signal levels (167).

Stochastic resonance is but one of the phenomena exhibited by neural systems driven by noise (8, 168). For instance, if a neuron receives a subthreshold periodic input, noise can elicit spike firings that tend to be locked to the input (13). This is because a small amount of noise will determine an output with the strongest signature of the periodic input. This effect depends on a linearization of the threshold by noise, and on disruption of phase locking.

Another related phenomenon is represented by the coherence resonance, which consists in noise-sustained oscillations at an optimal noise intensity. Moreover, noise can enhance the synchronization and pattern formation in excitable systems (8, 168).

## 9. Some clinical considerations

### 9.1. Aging

As we remarked above (see section 6.7), perceptual thresholds for motion discrimination tend to deteriorate in asymptomatic older persons. Vestibular function is affected by aging after the age of 40, about two decades earlier than visual acuity, odor discrimination, or speech intelligibility (107). It has been argued that this early functional decline may depend on the specific vulnerability of the vestibular apparatus to the action of free radicals (107). Vestibular afferents may be damaged by free radicals earlier than other sensory afferents because of their higher metabolic demands. Primary vestibular afferents and central vestibular neurons in primates have high resting discharge rates (9). High resting activity involves heavy metabolic loads, with extensive oxidative ATP production by the mitochondria. Mitochondria can contribute oxidative stress and free radicals, which may cause local damage to the vestibular system (107).

#### 9.1.1. Effects on postural stability

The decline of vestibular function with aging affects both motion perception and postural stability. In fact, multivariate analyses showed that roll tilt perceptual thresholds correlate robustly with age and performance in static balance tasks (169). For example, at 60 years of age, the chance of failing the most demanding postural condition (i.e., standing with eyes closed on foam support, which requires increased vestibular reliance) was about 5% for the participants with the lowest roll tilt thresholds, but it was about 80% for those with the highest roll tilt thresholds (169). The relevance of this finding is that it is known that failure in this demanding postural task correlates with a high risk of falls in daily life (170).

### 9.2. Vestibulopathies

Vestibular motion thresholds are also elevated in the peripheral and central vestibulopathies that determine hypofunction [see (4, 96)]. Vestibular hypofunction can be idiopathic, postsurgical, neoplastic, autoimmune, or it can result from Meniere’s disease or medication side effects. For instance, earth-vertical translation thresholds in patients with total bilateral labyrinthectomy (vestibular ablation) were up to 57 times greater than normal (55). In these patients, performance decrements were smaller for discrimination of motion directions (e.g., roll tilt) where other sensory cues (e.g., somatosensory) can contribute appreciably. Idiopathic bilateral vestibulopathy mainly affects lateral canal and utricular thresholds, while it may spare vertical canal and saccular function (171). In patients with unilateral vestibular nerve section, detection thresholds for yaw rotation were $\sqrt{2}$ higher than for healthy persons, as expected by assuming that the lesion halved the variance of both the signal and noise (172). In vestibular migraine (173) and persistent perceptual-postural dizziness (174) motion thresholds can be lower than in age-matched controls, indicating enhanced sensitivity of the vestibular apparatus.

As we remarked before (see sections 4, 5, and 7), healthy vestibular neurons at different stages of central pathways are tuned to natural head motion stimuli. Head motion is first sensed and then processed by central vestibulo-motor pathways. This results in a feedback loop that influences subsequent behavior, including head motion itself. Accordingly, loss of peripheral vestibular inputs in patients with chronic unilateral vestibular hypofunction alters the statistics of head movements during natural self-motion, especially for tasks that depend on rapid vestibular feedback, such as repetitive jumping or walking on foam (175).

As in the case of aging, altered motion thresholds in vestibular patients often lead to postural disturbances. It has been argued that increased noise in vestibular feedback caused by aging or pathology reduces the signal-to-noise ratio in vestibulo-spinal pathways, leading to increased sway, imbalance, and higher risk of falls (176).

### 9.3. Future promises from vestibular stimulation

In line of principle, vestibular stimulation interventions such as those reviewed above (see section 7) might be helpful to ameliorate either motion perception or postural instability in patients. However, current results are still insufficient to draw definitive conclusions. Thus, noisy GVS alone improved balance in bilateral vestibulopathies (137, 177). However, when it was combined with vestibular rehabilitation training in the same category of patients, it did not lead to any significant improvement relative to that obtained with vestibular rehabilitation alone (178).

### 9.4. Strong acoustic noise and vibrations

In this article, we mainly considered the effects of low to medium-intensity extrinsic noise. A separate issue is represented by the pathological effects of strong acoustic noise and vibrations, such as those present in heavy industry or military environment. Here, we only mention a few pertinent findings. Noise exposure higher than 90 dB for 8 h/day for prolonged periods of time can cause hearing loss as well as vestibular dysfunction (179–181). Moreover, canal deficits diagnosed with video head impulse test were found in workers exposed to daily, intense whole-body vibrations (182).

## 10. Conclusion

We considered some effects of both extrinsic (exogenous) and intrinsic (endogenous) noise on the perceptual responses and neural processing of self-motion signals at different stages of the vestibular pathways. Although noise can interfere with neural processing and performance, all vestibular neurons, starting from the mechanoceptors of the inner ear up to cortical neurons, exhibit built-in mechanisms that tend to limit the impact of noise, and in several cases to exploit noise so as to enhance sensitivity to naturalistic motion stimuli. We also showed that stochastic facilitation of neural processing and behavioral performance can be obtained experimentally by applying optimal levels of noisy external perturbations on top of subliminal motion stimuli. This is a potentially promising approach to be further explored for vestibular rehabilitation in case of vestibular hypofunction (e.g., in elderly people) or vestibulopathies.

## Author contributions

All authors listed have made a substantial, direct, and intellectual contribution to the work and approved it for publication.

## Funding

The experimental work performed in the authors’ laboratories was supported by the Italian Ministry of Health (Ricerca corrente, IRCCS Fondazione Santa Lucia, Ricerca Finalizzata RF-2018-12365985), Italian Space Agency (grant I/006/06/0), INAIL (BRIC 2019 BRISK and BRIC 2022 LABORIUS), and Italian University Ministry (PRIN grant 2020EM9A8X_003 and NRRP project MNESYS PE00000006). #NEXTGENERATIONEU (NGEU) National Recovery and Resilience Plan project A Multiscale integrated approach to the study of the nervous system in health and disease, DN. 1553 11.10.2022.

## Conflict of interest

The authors declare that the research was conducted in the absence of any commercial or financial relationships that could be construed as a potential conflict of interest.

## Publisher’s note

All claims expressed in this article are solely those of the authors and do not necessarily represent those of their affiliated organizations, or those of the publisher, the editors and the reviewers. Any product that may be evaluated in this article, or claim that may be made by its manufacturer, is not guaranteed or endorsed by the publisher.
